# DNA-methylation patterns imply a common cellular origin of virus- and UV-associated Merkel cell carcinoma

**DOI:** 10.1038/s41388-021-02064-1

**Published:** 2021-10-19

**Authors:** Jan Gravemeyer, Ivelina Spassova, Monique E. Verhaegen, Andrzej A. Dlugosz, Daniel Hoffmann, Anja Lange, Jürgen C. Becker

**Affiliations:** 1 Translational Skin Cancer Research (TSCR), University Duisburg-Essen, Essen, Germany; 2 German Cancer Consortium (DKTK) & German Cancer Research Center (DKFZ), Heidelberg, Germany; 3 Department of Dermatology, University Hospital Essen, Essen, Germany; 4 Department of Dermatology, University of Michigan, Ann Arbor, MI USA; 5 Department of Cell and Developmental Biology, University of Michigan, Ann Arbor, MI USA; 6 Bioinformatics & Computational Biophysics, University Duisburg-Essen, Essen, Germany

**Keywords:** High-throughput screening, Cancer of unknown primary, Cancer genomics

## Abstract

Merkel cell carcinoma (MCC) is a neuroendocrine tumor either induced by integration of the Merkel cell polyomavirus into the cell genome or by accumulation of UV-light-associated mutations (VP-MCC and UV-MCC). Whether VP- and UV-MCC have the same or different cellular origins is unclear; with mesenchymal or epidermal origins discussed. DNA-methylation patterns have a proven utility in determining cellular origins of cancers. Therefore, we used this approach to uncover evidence regarding the cell of origin of classical VP- and UV-MCC cell lines, i.e., cell lines with a neuroendocrine growth pattern (*n* = 9 and *n* = 4, respectively). Surprisingly, we observed high global similarities in the DNA-methylation of UV- and VP-MCC cell lines. CpGs of lower methylation in VP-MCC cell lines were associated with neuroendocrine marker genes such as *SOX2* and *INSM1*, or linked to binding sites of *EZH2* and *SUZ12* of the polycomb repressive complex 2, i.e., genes with an impact on carcinogenesis and differentiation of neuroendocrine cancers. Thus, the observed differences appear to be rooted in viral compared to mutation-driven carcinogenesis rather than distinct cells of origin. To test this hypothesis, we used principal component analysis, to compare DNA-methylation data from different epithelial and non-epithelial neuroendocrine cancers and established a scoring model for epithelial and neuroendocrine characteristics. Subsequently, we applied this scoring model to the DNA-methylation data of the VP- and UV-MCC cell lines, revealing that both clearly scored as epithelial cancers. In summary, our comprehensive analysis of DNA-methylation suggests a common epithelial origin of UV- and VP-MCC cell lines.

## Introduction

Merkel cell carcinoma (MCC) is an aggressive neuroendocrine tumor of the skin. It can be subdivided into two groups: virus-associated MCCs (VP-MCCs) in which carcinogenesis depends on the integration of the Merkel cell polyomavirus (MCPyV) and virus-negative MCCs that are driven by UV-light-induced mutations (UV-MCCs) [1]. The latter are characterized by a higher mutational burden and often carry mutations in *RB1* [2, 3]. Aberrations in the retinoblastoma (pRb) pathway are critical for MCC development since its disruption not only leads to deregulation of the cell cycle but also induces *SOX2* expression thereby causing neuroendocrine transformation [4]. In VP-MCCs, pRb functions are repressed by binding of MCPyV-encoded large T-antigen (LT) [5]. Thus, VP- and UV-MCCs deregulate the same key pathway but by different means.

The cellular origin of MCC is unknown. Besides B cells and Merkel cells, the most prominent hypothesis discusses a mesenchymal origin of VP-MCCs and an epithelial origin of UV-MCCs [6, 7]. UV-light exposure of keratinocytes explaining the accumulated UV-light mutations and reports of mixed squamous cell carcinoma (SCC)/UV-MCC tumors argue toward an epithelial origin of UV-MCC [8, 9]. Recently, whole exome sequencing of combined tumors consisting of SCC in situ and MCPyV-negative MCC demonstrated that many mutations were shared between SCC and MCC, thus indicating a common ancestry [10]. Fibroblasts, which are not exposed to UV-light, are an appealing candidate for the origin of VP-MCCs since they are to date the only cell type able to support the MCPyV’s life cycle [11, 12]. However, for other polyomaviruses, abortive infections, such as blocked viral replication due to cell type specific differences, favor cell transformation [13]. Moreover, MCPyV’s early transforming genes together with an experimental *GLI1* expression induce a Merkel cell like differentiation in epithelial cells and a mixed trichoblastoma/VP-MCC has been described [14, 15].

Although DNA methylation changes dynamically during tumor cell differentiation, it retains an epigenetic memory of the cancer’s cell of origin and has therefore been frequently exploited for cell lineage classification [16, 17]. However, these marks must be differentiated from transformation-specific changes of the epigenome. Here, we examined the DNA-methylation of classical VP- and UV-MCC cell lines to compare their cellular origins. Surprisingly, the DNA-methylation patterns of VP- and UV-MCC cell lines revealed very few differentially methylated regions (DMRs), which are mainly associated with genes involved in the neuroendocrine transformation of cancers. Furthermore, we established a DNA-methylation-based score for epithelial characteristics revealing a similar score for VP- and UV-MCC cell lines.

## Results

### Comparable DNA-methylation profiles of VP-MCC and UV-MCC cell lines

**Fig. 1:**
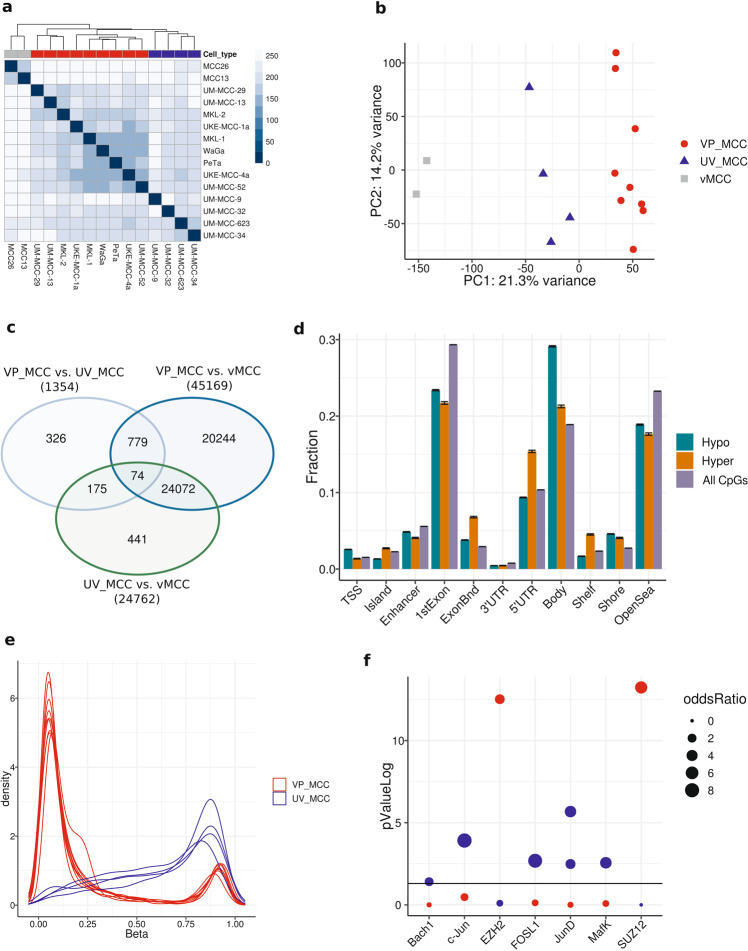
Similar DNA-methylation pattern in VP- and UV-MCC cell lines. **a** Hierarchical clustering (ward.D2, euclidean distance) based on DNA-methylation using beta values of all CpGs. The dendrogram shows the clustering of vMCC cell lines (gray), VP-MCC (red), and UV-MCC (blue). The heatmap refers to the pairwise distances in DNA-methylation between cell lines. The score expresses the amount of differences between samples. A score of 0 (dark hue) indicates identical methylation patterns., which is true for the diagonal, reflecting the distance of a cell line to itself. The higher the score the greater the difference. **b** Principal component analysis using the same cell lines and methylation data as in (**a**). Percentages on the axes refer to the extent of variation explained by each component. Depicted are the first two components explaining the largest spread in DNA-methylation. **c** Venn diagram showing overlaps of pairwisely calculated DMPs for three comparisons: VP-MCC vs. UV-MCC, VP-MCC vs. vMCC and UV-MCC vs. vMCC cell lines. The sum of the numbers from the respective segments of an ellipse refers to the amount of identified DMPs in a comparison, the numbers in the segments refer to coinciding DMPs identified in either comparisons. **d** Annotation of genomic features of the 1354 DMPs between VP- and UV-MCC cell lines from (**c**). CpGs were mapped to their corresponding elements in the genome. Hypo (turquoise) and hyper (orange) refer to CpGs that are hypo- or hypermethylated in VP-MCC compared to UV-MCC cell lines. The distribution of genomic features of all CpGs on the EPIC array (purple) is shown to place the observed differences. Error bars are 95% confidence intervals of 1000 bootstrapping iterations. **e** Density distributions of beta values using the 1354 DMPs from (**c**). Each line refers to a single MCC cell line. The degree of methylation is represented by the beta value on the *x*-axis while relative frequencies of DMPs are expressed as densities on the *y*-axis. A beta value of 1 means fully methylated and a value of 0 unmethylated. **f** Enrichment of ChIP-seq peaks for transcription factor (TF)-binding using H1 human embryonic stem cell data. Reference data were taken from the ENCODE database and applied on the 1354 DMPs between VP- and UV-MCC cell lines using Locus Overlap Analysis (LOLA). TF-binding enrichments for sites that have lower methylation in VP-MCC cell lines are depicted in blue, with lower methylation in UV-MCC cell lines in red. The *y*-axis shows the log_10_ transformed *p* value with the horizontal black line cutting at 0.05. Dot sizes indicate the effect size of the enrichment expressed as odds ratios. LOLA results are summarized in Supplementary Table 3, including confidence intervals for the odds ratios.

DNA-methylation has high tissue specificity and has proven valuable to identify the origin of cancers [18–20]. In an attempt to identify the cells of origin of viral- and UV-associated MCCs, we established the DNA-methylation patterns of VP- and UV-MCC cell lines using the EPIC array comprising about 850,000 CpGs (Supplementary Table 1). Hierarchical clustering (ward.D2, euclidean distance) and principal component analysis (PCA) of the DNA-methylation data demonstrated that classical VP-MCC cell lines are more similar to each other than to classical UV-MCC cell lines (Fig. 1a, b). However, the gap between classical VP- and UV-MCCs is smaller than to the variant MCC cell lines (vMCCs) MCC13 and MCC26 (Fig. 1b). vMCCs are characterized by an adherent growth pattern, i.e., they display incomplete neuroendocrine properties [21]. We included vMCC cell lines for analysis to put differences between classical VP- and UV-MCCs into perspective. Indeed, they clustered apart from UV- and VP-MCC cell lines in hierarchical clustering and PCA space (Fig. 1a, b). Alikeness of UV- and VP-MCCs was also reflected by the respective numbers of differentially methylated CpG probes (DMPs). We called DMPs for three comparisons: VP-MCCs vs. UV-MCCs, VP-MCCs vs. vMCCs, and UV-MCCs vs. vMCCs using *p* value ≥ 0.01 and log_2_FC ≥ 2 as criteria. Only 1354 DMPs were identified between VP- and UV-MCC cell lines, whereas 45,169 and 24,762 DMPs were observed in the comparison of vMCC with VP- and UV-MCC cell lines, respectively (Fig. 1c). Thus, at least about 20-fold less DMPs were observed comparing UV- with VP-MCC cell lines than comparing either of them with vMCC cell lines. Of note, 24,072 DMPs overlapped between vMCC vs. UV- and VP-MCC cell lines. Since DMPs can be located in regions of the genome without strong effects on gene regulation, we annotated the genomic location of the observed DMPs between VP- and UV-MCC cell lines (Fig. 1d and Supplementary Table 2). For this, DMPs were split into sites of hypo- and hypermethylation in VP- compared to UV-MCC cell lines. The EPIC array provides an excerpt of about 850,000 CpGs, which may result in biased proportions of CpGs located in, e.g., CpG island compared to the whole genome. To address this notion, we visualized the proportions of genomic regions probed by the array by calculating the overall genomic distribution of all CpGs on the EPIC array (Fig. 1d). An increased fraction of DMPs was located at transcription start sites (TSS) and in CpG shores that occur in short distance to CpG-islands but were less frequent in intergenic regions (OpenSea) and in the first exon of genes. An enrichment of DMPs in CpG shores and depletion in intergenic regions has also been reported for HPV-positive and -negative head and neck squamous cell carcinoma (HNSCC) [22]. Hypermethylated sites occurred more often in the 5’UTR and in exon boundaries, whereas hypomethylated sites were more frequently situated in gene bodies (exons/introns). Most of the identified DMPs resulted from lower methylation in VP-MCC than in UV-MCC cell lines comparable to HPV-positive and -negative HNSCC (Fig. 1e) [22]. It should be noted that CpGs with a beta value of 0 are unmethylated and those with a value of 1 are fully methylated. To scrutinize whether the identified DMPs are indicative of differences in transcription factor (TF) binding we used the Locus Overlap Analysis (LOLA) to test for enrichments in TF-binding sites based on ChIP-seq peaks from the Encyclopedia of DNA Elements (ENCODE) database (Fig. 1f and Supplementary Table 3). Sites of lower methylation in VP-MCC cell lines were enriched for SUZ12 and EZH2 binding sites with a *p* value of 5.9 ∙ 10^−14^ and 5.6 ∙ 10^−13^ and odd ratios of 5.3 [CI_95%_ = 3.7–7.6] and 2.7 [CI_95%_ = 2.1–3.5], respectively; both TFs belong to the polycomb repressive complex 2, mediating transcriptional silencing by histone methylation [23]. DMPs hypomethylated in UV-MCC cell lines were enriched for binding sites of c-Jun (JUN), FOS like 1 (FOSL1), and JunD (JUND). All three proteins are subunits of the AP-1 TF complex [24].

### *SOX2* and *INSM1* are hypermethylated in UV-MCC cell lines

**Fig. 2:**
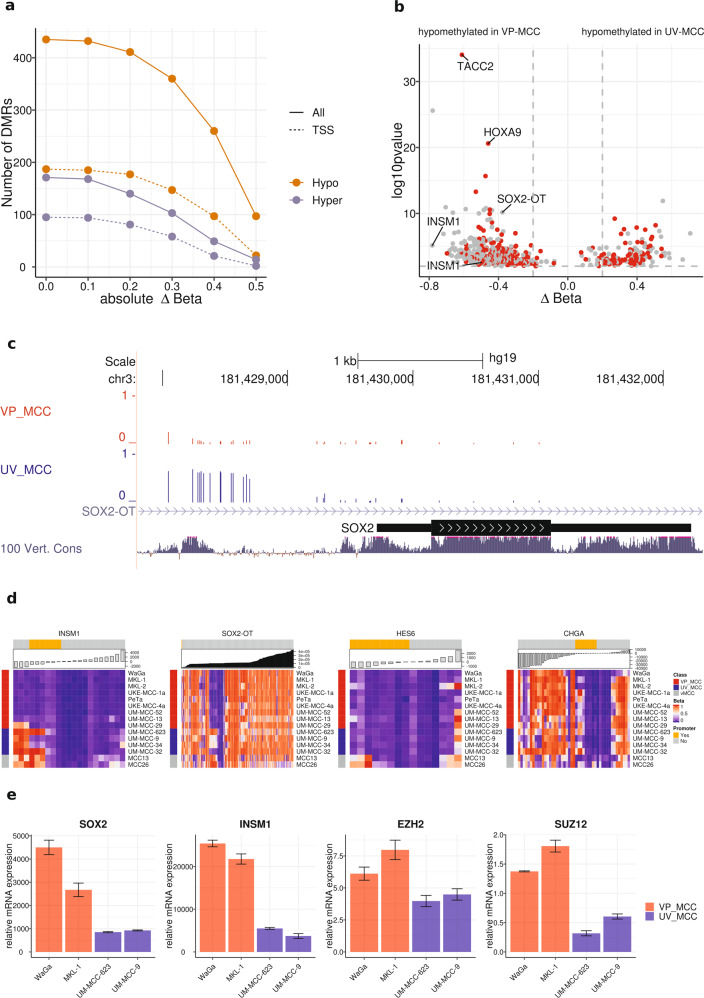
DNA methylation of *SOX2* and *INSM1* varies between VP- and UV-MCC cell lines. **a** Dependence of the number of identified DMRs (*p* value ≤ 0.01) on the effect size (absolute ΔBeta) between UV- and VP-MCC cell lines. “Absolute ΔBeta” is the difference of the mean beta values of all CpGs in a DMR in absolute terms. The total of 606 DMRs was split into DMRs of lower (orange) and higher (purple) methylation in VP- compared to UV-MCC cell lines. Solid lines represent the total number of DMRs and dashed lines only those located near the TSS. **b** Volcano plot of DMRs between VP- and UV-MCC cell lines. The *y*-axis shows the negative log_10_ transformed *p* values and the *x*-axis the mean difference in methylation (ΔBeta). Negative differences indicate lower methylation in VP-MCC cell lines, positive differences indicate lower methylation in UV-MCC cell lines. DMRs marked by red dots are located within 2000 bp upstream of the TSS of a gene. **c** The SOX2-OT locus visualized in the UCSC genome browser. The upper two barplot tracks show CpG methylation in VP-MCC (red) and UV-MCC (blue) cell lines. Each bar represents a single CpG site and bar height the beta value. The thin blue line is the SOX2-OT intron and the dark black box the SOX2 exon inside the intron. The “100 Vert. Cons” track indicates sequence conservation across vertebrates and is indicative of functional regions in the differentially methylated CpG cluster upstream of the SOX2 TSS. **d** Heatmaps of methylation levels (beta values) of all CpGs associated with *INSM1*, *SOX2-OT*, *HES6* and *CHGA*. Columns represent CpG sites sorted by their distance to the TSS, which is also shown by the top barplots. CpGs within 2000 bp upstream of the TSS are colored in orange to indicate potential promoter associations. Cell lines are un-clustered as rows and color coded; VP-MCC (red), UV-MCC (blue), and vMCC (gray) cell lines. **e** Relative mRNA expression of *SOX2*, *INSM1*, *EZH2*, and *SUZ12* in the VP-MCC cell lines WaGa and MKL-1 as well as the UV-MCC cell lines UM-MCC-623 and UM-MCC-9 measured by qPCR. Expression was normalized to fibroblast cell lines. Error bars refer to the standard deviation of the qPCR measurements.

Since methylation usually affects neighboring CpG sites in the same manner, we combined nearby sites into DMRs (≥3 CpGs, in a 1000 bp window) to reduce complexity of the data. Thereby, we observed 606 DMRs of which 171 were hyper- and 435 hypomethylated in VP-MCC compared to UV-MCC cell lines (*p* value ≤ 0.01, Fig. 2a). Only half of them were located within 2000 bp upstream of a TSS, i.e., with potential implications for the promoter. Harsher filtering by differential methylation (Δ*β*_mean_ ≥ [0.1, 0.2, 0.3, 0.4, 0.5]) did not affect the ratio of hyper- to hypomethylated DMRs or their relative proportions in TSS regions (Fig. 2a and Supplementary Table 4).

Hypomethylated DMRs with TSS annotation mapped to the transforming acidic coiled-coil containing protein 2 (*TACC2*), and homeobox A9 (*HOXA9*) (Fig. 2b and Supplementary Table 5). *HOXA9* is a developmental regulator of the homeobox group [25]. *TACC2* is a centrosome and microtubule interacting protein, which is targeted by Simian virus 40 LT-antigen to disrupt microtubule organization [26]. Importantly, hypomethylated DMRs were also located in the TSS of *INSM1* (Insulinoma-associated 1) and the *SOX2-OT* (SOX2 Overlapping Transcript). It should be noted that the *SOX2-OT* DMR is located in short distance to the TSS of the actual *SOX2* TF nested within the *SOX2-OT* intron (Fig. 2c). *SOX2* and *INSM1* are frequently expressed in MCC and other neuroendocrine tumors and are regarded as markers for a neuroendocrine phenotype [27–29]. Analysis of all CpGs with annotations for *INSM1* and *SOX2-OT* revealed that hypermethylation was evident in all UV-MCC and vMCC cell lines (Fig. 2d). However, TSS regions of other neuroendocrine marker genes, i.e., *HES6* and *CHGA*, had equally low methylation in VP- and UV-MCC cell lines (Fig. 2d). This, however, was not the case for vMCC cell lines in which the *HES6* and *CHGA* TSS region was hypermethylated.

To test if higher DNA-methylation levels influence gene expression, qPCRs were performed using UM-MCC-623 and UM-MCC-9 with high SOX2 DNA-methylation as well as WaGa and MKL-1 with low DNA-methylation levels. Indeed, *SOX2* and *INSM1* were expressed in all but in lesser amounts in UV-MCC cell lines (Fig. 2e). In addition, we measured gene expression of *EZH2* and *SUZ12* for which differential TF-binding was inferred by LOLA; both genes, particularly *SUZ12*, were lower expressed in UV-MCC cell lines (Figs. 1f and 2e).

### VP- and UV-MCC cell lines share neuroendocrine and epithelial DNA-methylation patterns

**Fig. 3:**
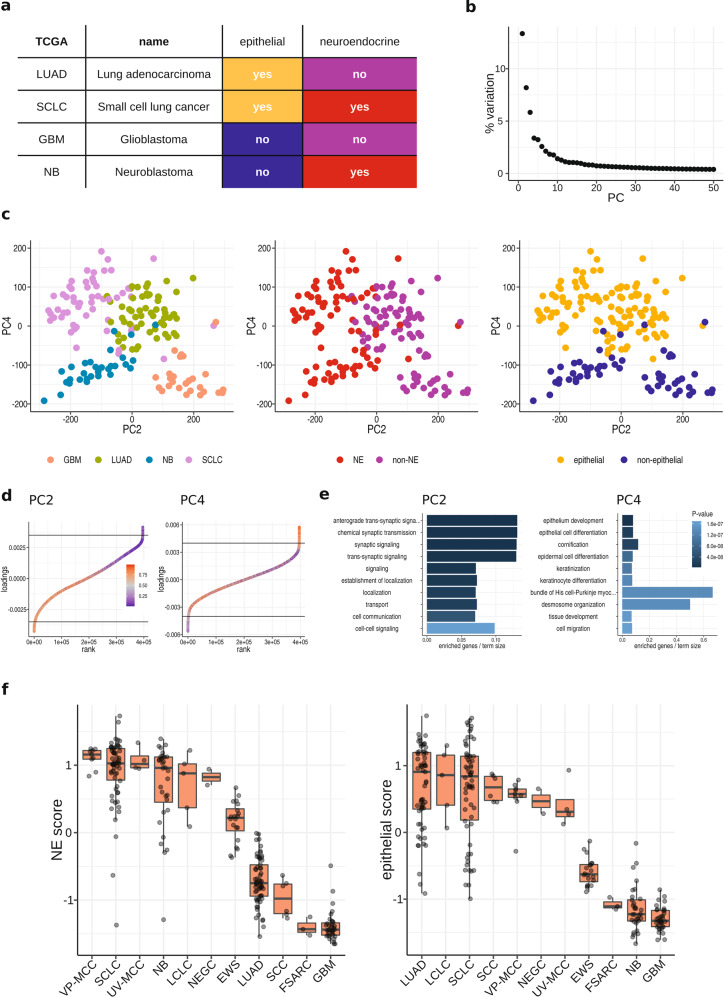
Epithelial properties of VP- and UV-MCC cell lines. **a** Table of cancer types for which publicly available DNA-methylation data were used. The coloring highlights pairs of neuroendocrine or epithelial cancers. **b** PCA on DNA-methylation beta values of SCLC, LUAD, NB, and GBM cell lines from (**a**). Shown is the amount of variation explained by each component independently in percent. The elbow at about 10 components indicates that later components are less informative. **c** PCA plot corresponding to (**b**) using PC2 and PC4. Each dot belongs to a single cell line and coloring refers to the cancer entities (left panel), neuroendocrine cancers (middle panel), or epithelial cancers (right panel). **d** CpGs ranked by their loadings of PC2 (left) and PC4 (right). Most informative CpGs are located at the curve tails, which show a sharp increase in loading values (indicated by black horizontal lines). The color code reflects the mean beta value for DNA-methylation of CpGs averaged over all neuroendocrine cancer cell lines (NB and SCLC) for PC2 and all epithelial cancer cell lines (LUAD and SCLC) for PC4. A strong blue coloring means that CpGs are low on methylation (vice versa for red coloring). **e** Gene Ontology analysis based on genes that correspond to CpGs selected in (**d**). Enrichment was performed independently for genes associated with PC2 (left) and genes associated with PC4 (right). **f** Neuroendocrine score based on PC2 (left panel) and the epithelial score (right panel) based on PC4. The scoring model was calculated by using CpG loadings selected in (**d**) and projected onto additional cell lines. Scores were *Z*-score normalized and higher values are associated with increased neuroendocrine or epithelial properties. Boxplots summarize the score distributions per cancer type (EWS Ewing’s sarcoma, FSARC fibrosarcoma, GBM glioblastoma, LCLC large cell lung cancer, LUAD lung adenocarcinoma, NEGC neuroendocrine gastric carcinoma, NB neuroblastoma, SCC squamous cell carcinoma, SCLC small cell lung cancer, UV-MCC UV-light-associated MCC, VP-MCC virus-associated MCC); each dot represents a single cell line.

To relate the observed DNA-methylation patterns to cellular characteristics, we collected DNA-methylation data from small cell lung cancer (SCLC), lung adenocarcinoma (LUAD), glioblastoma (GBM), and neuroblastoma (NB) cell lines (Fig. 3a and Supplementary Table 6). SCLC and LUAD are epithelial cancers from the lung, with SCLC expressing a neuroendocrine phenotype [30, 31]. NB and GBM originate from the nervous tissue with NB displaying a neuroendocrine phenotype [32, 33]. These pairings have been used before to scrutinize the carcinogenesis of neuroendocrine cancers [34]. Next, we performed PCA on the beta values for DNA-methylation to investigate principal components (PCs) summarizing the most variation between the cell lines (Fig. 3b). While PC1 was associated with sample specific variations, PC2 and PC4 represented the top components that stratified the samples by neuroendocrine and tissue properties. Specifically, PC2 separated the cell lines into neuroendocrine (NB and SCLC) and non-neuroendocrine (GBM and LUAD) (Fig. 3c), whereas PC4 stratified them into epithelial (SCLC and LUAD) and non-epithelial (NB and GBM) cancers.

In a PCA, each observation (here the CpG methylation status) is weighted according to its contribution to each component. The weights (loadings) were used to develop a neuroendocrine and an epithelial PC score. Specifically, the highest weights were selected using rank plots to reduce both the high dimensionality of the data and cumulative noise effects from less informative CpGs (Fig. 3d). Only CpGs in the steepest part of the rank plots were kept, yielding 2976 CpGs for PC2 and 2230 CpGs for PC4 (Supplementary Table 7). PCA explains variation in the data as superposition of variations in linearly independent composite directions, the principle components. Different PCs thus essentially represent independent biological programs [35]. In order to interpret PCs of the DNA-methylation biologically, we performed Gene Ontology (GO) analysis of genes associated with the selected CpGs. It demonstrated that variation in PC2 is related to neuroendocrine vs. non-neuroendocrine pathways and PC4 is related to epithelial vs. non-epithelial pathways (Fig. 3e). Subsequently, CpG loadings were used to weight beta values and to infer a neuroendocrine and an epithelial score. As expected, neuroendocrine scores were high for SCLC and NB but low for the non-neuroendocrine LUAD and GBM (Fig. 3f). Conversely, the epithelial score was high for SCLC and LUAD but low for NB and GBM originating from nervous tissue (Fig. 3f). The model was validated by projecting the scoring on further cell lines including large cell lung carcinoma (LCLC), cutaneous squamous cell carcinoma (cSCC), neuroendocrine gastric carcinoma (NEGC), Ewing’s sarcoma (EWS), and fibrosarcoma (FSARC) as it correctly identified their characteristics. LCLC is epithelial and neuroendocrine, cSCC develops from keratinocytes, and both FSARC and EWS cell lines originate from primitive mesenchymal cells [30, 36–41]. Most importantly, projection on VP- and UV-MCC cell lines not only revealed a high neuroendocrine score for either group, comparable to SCLC and NB, but both also displayed a high epithelial score, which was comparable to that of SCC, SCLC, and LCLC.

## Discussion

The cell of origin of MCC is unknown. In fact, VP- and UV-MCCs may originate from different cells or even different tissue types [6, 7]. Recent observations indicate that cancer epigenomes, particularly DNA-methylation marks, result from both, transformation-specific changes and epigenetic patterns already present in the cell of origin that was transformed into a neoplastic cell [18, 42]. Comparison of DNA-methylation patterns of VP- and UV-MCC cell lines revealed only 1354 DMPs, which seems few compared to the differences with the vMCC cell lines. Most of these DMPs were located close to actual gene structures, e.g., in exons or CpG shores, suggesting a more dynamic regulation of gene expression as opposed to more stable differences in DNA-methylation of enhancer regions associated with cellular origins [43]. The depletion of intergenic DMPs between VP- and UV-MCC cell lines is consistent with comparisons of HPV-positive and -negative HNSCC tumors where DMPs occurred were less common in open sea regions but more prevalent in CpG shores [22].

The phenotypic characteristics of MCC, irrespective of its viral or UV-associated carcinogenesis, result from cell cycle deregulation and neuroendocrine differentiation. Cell cycle deregulation in VP- and UV-MCCs is achieved by mutations inactivating *RB1* or LT-pRb interactions. pRb repression also results in upregulation of SOX2 and the SOX2-target ATOH1 [3–5]. ATOH1 expression is crucial for neuroendocrine differentiation [21, 44, 45]. However, ATOH1 signaling is partially compensated by a repressor complex that includes INSM1, modulating neuroendocrine transformation [45, 46]. Most of the DNA-methylation variations between VP- and UV-MCCs relate to differences in the deregulation of pathways during viral- or UV-associated carcinogenesis. This includes higher DNA-hypermethylation of *SOX2* and *INSM1* in UV-MCCs, but also association of more hypomethylated DMPs in VP-MCCs at EZH2 and SUZ12 DNA binding sites. The latter are chromatin remodelers associated with neuroendocrine cancers [47–49]. The methylation-dependent repression of *TACC2* and *HOXA9* in UV-MCC cell lines is likely to be achieved in VP-MCC cell lines by other means; the Simian virus 40 (SV40)-encoded LT inhibits TACC2 protein function and *HOXA9* is a target of EZH2/SUZ12-based repression [26, 50–52]. Therefore, it is presumed that the observed variations in DNA-methylation between UV- and VP-MCCs are due to their respective forms of carcinogenesis rather than to distinct cells of origins. Since DNA-methylation patterns of cancer is the sum of both the processes causing transformation and the cell of origin being transformed, we aimed to distinguish the respective contribution to the observed patterns. Specifically, we addressed the impact of neuroendocrine transformation since it can be assumed to be the most relevant confounder [42]. Specifically, PCA of DNA-methylation data from neuroendocrine and non-neuroendocrine tumor cell lines derived from epithelial or non-epithelial tissues was used to develop an epithelial and a neuroendocrine score. Projection of this model on the DNA-methylation data from MCC cell lines revealed high neuroendocrine and epithelial scores for both VP- and UV-MCC cell lines. We validated these scores with a number of epithelial, neuroendocrine (SCLC, LCLC, NEGC) and mesenchymal (FSARC, EWS) cancer cell lines. Only for EWS, did we observed a higher neuroendocrine score than expected; however, EWS tumors are composed of small round cells, like neuroendocrine cancers, and express neuronal marker genes such as neuron specific enolases [53].

DNA-methylation is dynamic, but some DNA-methylation patterns may be retained as a form of epigenetic memory. Indeed, DNA methylation—particularly in enhancer regions—may constitute a stable epigenetic mark inherited through multiple cell divisions [54]. Thus, DNA-methylation profiles can be useful for lineage classification. Here, by distinguishing factors influencing epigenetic patterns such as neuroendocrine transformation from assumed epigenetic marks characteristic for the cell of origin, we demonstrate that both VP- and UV-MCC cell lines can be expected to be of epithelial origin. In that sense, our data are in agreement with a recent case report of demonstrating by mutational overlap that a VP-MCC was derived from a trichoblastoma, i.e., suggesting an epidermal origin in the hair follicle [14]. Moreover, it has been demonstrated for adenocarcinomas of lung and prostate that epithelial cancers can be transformed into their neuroendocrine counterparts by dual inhibition of *RB1* and p53 [55]. For MCC, overexpression of ATOH1 in vMCC cell lines, which are more similar to SCC than classical MCC cell lines, induced a neuroendocrine growth pattern [21, 45]. Thus, the epithelial DNA-methylation signature for VP- and UV-MCC cell lines complements the accumulating evidence of an epithelial origin of MCC independent on viral- or UV-associated carcinogeneis. It should be noted, however, that it is beyond the scope of this work to completely rule out the possibility that neuroendocrine transformation causes the methylomes of VP- and UV-MCCs to converge.

Possible limitations of our work could be seen in our focus on analyzing cell lines. First of all, although the number of scrutinized cell lines is rather large compared to other studies in MCC, it is still limited. Secondly, the direct use of tumor samples would certainly have advantages; however, cell lines are extremely useful for determining cell origin patterns as they do not suffer from sample impurity. Two recent studies addressed differences in DNA-methylation between VP- and UV-MCCs using either MCC tissue or cell lines [56, 57]. Consistent with the here reported results, both studies found a relatively low number of DMPs (470 and 2260) with the majority of DMPs being hypomethylated in VP-MCC samples in these reports as well. However, neither group inferred tissue of origin signatures since clustering of MCC with samples from dermis, epidermis, and nerve tissues resulted in a clear separation of MCC tissues or because MCC cell lines clustered together with other neuroendocrine entities [56, 57]. The PCA derived scoring of the present study allowed conclusion on the tissue of origin by deconvolution of the data into its underlying components, exploiting the purity of cell lines.

In summary, our data strongly suggest that the minor variations in the methylation pattern of VP- and UV-MCC subtypes are due to differences of viral- or UV-associated carcinogenesis rather than of different cells of origin.

## Methods

### Cell lines

The cell lines WaGa, PeTa, MKL-1, MKL-2, UKE-MCC-1a, UKE-MCC-4a, MCC13, and MCC26 were maintained in RPMI-1640 (Pan Biotech, Aidenbach, Germany) supplemented with 10% fetal calf serum (Sigma, St. Louis, MO, USA) and 1% penicillin/streptomycin (Pan Biotech, Aidenbach, Germany), for UM-MCC-9, UM-MCC-13, UM-MCC-29, UM-MCC-32 and UM-MCC-34, UM-MCC-52, and UM-MCC-623 the medium was supplemented with 15% chicken embryonic extract [58]. The SCC cell lines Met-1, Met-4, SCL-1, SCL-2, SCC-13, HSC-1 were maintained in DMEM supplemented with 10% fetal calf serum and 1% penicillin/streptomycin [21]. Due to the rareness of MCC we included all available cell lines. An overview of the cell lines used is given in Supplementary Table 1.

### qPCRs

Gene expression was measured by qPCR using the SYBR green assay (Sigma-Aldrich, St. Louis, Missouri, USA, L6544-500RXN). Relative quantification was calculated using the ΔΔCt method and normalized to a fibroblast cell line. mRNA expression was measured for *EZH2* (fw: GACCTCTGTCTTACTTGTGGAGC, rev: CGTCAGATGGTGCCAGCAATAG), *SUZ12* (fw: CCGAGCACTGTGGTTGAGTA, rev: AACTGCATCTGATGGTGGTG), *INSM1* (fw: ATTGAACTTCCCACACGA, rev: AAGGTAAAGCCAGACTCCA), and *SOX2* (fw: GCTTAGCCTCGTCGATGAAC, rev: AACCCCAAGATGCACAACTC). *HPRT* served as endogenous control (fw: GTCGTGATTAGTGATGATG, rev: GTTCAGTCCTGTCCATAA).

### DNA methylation

DNA methylation was measured using the Infinium MethylationEPIC BeadChip array (Illumina, Ense-Höingen, Germany), which covers about 850,000 CpGs sites. For this, DNA from all cell lines was isolated using Qiagen QiAamp DNA Mini Kit following the manufacturer’s instructions. The EPIC array analysis was performed at the DKFZ Genomics and Proteomics Core Facility. Afterwards, raw IDAT files were processed in R version 3.5.3 using the minfi cross-package workflow [59] as described previously [21]. We applied functional normalization to deal with technical variations and used PCA to test for batch effects. CpG probes were discarded from the data set if the detection *p* value was above 0.01 in at least one sample. Furthermore, we removed probes that were located on sex chromosomes, showed cross-reactivity, or have a SNP at the same site as the CpG. A total of 761,251 CpGs remained after filtering. For visualization, clustering and PCA, the methylation (M) and unmethylation (U) signals were expressed as beta values $\left( {\beta = \frac{M}{{M + U + 100}}} \right)$. To identify DMPs, beta values were logit transformed into M-values and called using the limma fit and eBayes functions (*p* value ≤ 0.01, |log_2_FC| ≥ 2) [60]. DMRs were called using the dmrcate function (*p* value ≤ 0.01, at least 3 CpGs in 1000 bp distance) [59]. Differences in methylation levels of DMRs between VP- and UV-MCCs were summarized using the differences of mean beta values within a region (meanbetafc column after running dmrcate). Hierarchical clustering was performed using the hclust function (ward.D2, euclidean distance) and beta values. The hierarchical clustering recursively merges cell lines with similar DNA-methylation patterns into clusters depending on their euclidean distances. In case of just two CpGs {X, Y} and two cell lines {1, 2}, the euclidean distance would be Distance = [(X_1_ – X_2_)^2^ + (Y_1_ – Y_2_)^2^]^1/2^. Accordingly, with >2 CpGs it is obtained by the sum of the differences between each CpG as defined above. A higher distance value thus indicates greater dissimilarity in DNA-methylation between cell lines. In addition to the dendrogram in Fig. 1a, we also plotted these underlying distances between cell lines to visualize the amount of differences. PCA was performed using beta values and the prcomp function of the R stats package in its default settings (version 3.5.3). Genomic annotations (e.g., CpG Island, TSS, etc.) refer to the hg19 coordinates supplied by Illumina. These annotation were also used to annotate DMPs and calculate their frequencies in genomic regions (Fig. 1d). In order to obtain errors on the frequencies of genomic features of DMPs shown in Fig. 1d, we performed bootstrapping with 1000 iterations for the hypo, hyper, and EPIC set. The latter refers to the proportion of genomic features using all CpGs of the EPIC array.

### LOLA

The LOLA enables enrichment analysis of genomic regions comparable to gene-based enrichments of widely used gene set enrichment tools [61]. Here, we used the LOLA R package in combination with ChIP-seq data from the ENCODE database to test for enrichment of TF-binding sites in differentially methylated sites between UV- and VP-MCC cell lines (Fig. 1f). Since the cell of origin of MCC is unclear, we used ChiP-seq data from H1 human embryonic stem cells as reference. Indeed, we observed enrichments of, e.g., EZH2 and SUZ12 also in other cell types and found vice versa more TFs using all cell types available as reference. However, we decided on h1-ESC cells as a fixed reference to minimize assumptions on the cell of origin. Enrichments for all cell types of the ENCODE database with *p* value ≤ 0.05 are available in Supplementary Table 3. As background we provided all CpGs present on the EPIC array. Outside of LOLA, we additionally calculated 95% confidence intervals for the odds ratios using the contingency tables provided by LOLA and Fisher’s exact test to provide uncertainty estimates for LOLA enrichments (Supplementary Table 3).

### Principal component analysis-derived scores

DNA-methylation data were obtained from the GSE68379 GEO repository. Raw IDAT files of the Illumina 450k array from SCLC, LUAD, NB, GBM, NEGC, FSARC, EWS, and LCLC cell lines were downloaded and merged with self-generated EPIC array data using the combineArrays function of the minfi package [59]. Then, cell lines were normalized and filtered as described above. A list of the downloaded samples can be found in Supplementary Table 6. PCA of SCLC, LUAD, NB, and GBM was performed on scaled beta values using the prcomp R function of the default stats package. Loadings of PC2 and PC4 were plotted against their ranks and high loading CpGs extracted from the steeper parts of the curves (2976 CpGs for PC2, 2230 CpGs for PC4, Supplementary Table 7). For GO testing on the selected CpGs, the gometh function of the missMethyl package was used, which maps CpGs to gene IDs and performs pathway enrichment tests [62]. To derive a score that can also be projected onto other samples, beta values that correspond to the selected CpGs of either PC2 or PC4 were multiplied by their loadings. The loading adjusted beta values in each sample were summed up and *Z*-score normalized. In the case of PC2, loadings were multiplied by minus 1 before scoring to adjust for the direction of PC2.

## Supplementary information

Supplementary Table 1

Supplementary Table 2

Supplementary Table 3

Supplementary Table 4

Supplementary Table 5

Supplementary Table 6

Supplementary Table 7

## Data Availability

Datasets related to this article can be found at GSE178155 hosted at the Gene Expression Omnibus (GEO).

## Code availability

Data analysis and visualization was conducted using R (version 3.5.3) in conjunction with the packages specified in the Methods section. The R code is available upon request.
