# Supplementary material for: DNA-methylation patterns imply a common cellular origin of virus- and UV-associated Merkel cell carcinoma
Source: Oncogene. 2021 Oct 19;41(1):37–45. doi: 10.1038/s41388-021-02064-1 (PMC8724008; doi:10.1038/s41388-021-02064-1)
Supplement: Supplementary file 1 — Supplementary Table 1 [file 41388_2021_2064_MOESM1_ESM.docx]

| **Name** | **Type** | **Growth pattern** | **MCPyV status** | **Culture medium** | **References** |
| --- | --- | --- | --- | --- | --- |
| MCC13 | vMCC | Adherent | Neg | RPMI | (Leonard et al., 1995) |
| MCC26 |  | Adherent | Neg | RPMI | (Van Gele et al., 2002) |
| MKL-1 | VP-MCCs | Suspension, aggregates | Pos | RPMI | (Rosen et al., 1987) |
| MKL-2 |  | Suspension, aggregates | Pos | RPMI | (Van Gele et al., 2002) |
| PeTa |  | Suspension, aggregates | Pos | RPMI | (Houben et al., 2013) |
| WaGa |  | Suspension, aggregates | Pos | RPMI | (Houben et al., 2010) |
| UKE-MCC-1a |  | Suspension, aggregates | Pos | RPMI | Established in our lab |
| UKE-MCC-4a |  | Suspension, aggregates | Pos | RPMI | Established in our lab |
| UM-MCC-52 |  | Suspension, aggregates | Pos | CEM | (Czech-Sioli et al., 2020) |
| UM-MCC-29 |  | Suspension, aggregates | Pos | CEM | (Verhaegen et al., 2014) |
| UM-MCC-13 |  | Suspension, aggregates | Pos | CEM |  |
| UM-MCC-9 | UV-MCCs | Suspension, aggregates | Neg | CEM |  |
| UM-MCC-32 |  | Suspension, aggregates | Neg | CEM |  |
| UM-MCC-34 |  | Suspension, aggregates | Neg | CEM |  |
| UM-MCC-623 |  | Suspension, aggregates | Neg | CEM |  |
| HSC-1 | SCC | Adherent | Neg | DMEM | (Cheung et al., 2012) |
| Met-1 |  | Adherent | Neg | DMEM |  |
| Met-4 |  | Adherent | Neg | DMEM |  |
| SCC-13 |  | Adherent | Neg | DMEM | (Rheinwald and Beckett, 1981) |
| SCL-1 |  | Adherent | Neg | DMEM | (Reichrath et al., 2004) |
| SCL-2 |  | Adherent | Neg | DMEM |  |

**Supplementary table 1. Overview about cell lines**

Cell lines used for the Illumina EPIC array annotated by their cell line groups, MCPyV status and the culture medium. CEM stands for chicken embryonic extract medium and refers to chicken serum generated from the embryos.

**Supplementary References**

Cheung BB, Koach J, Tan O, Kim P, Bell JL, D'andreti C, et al. The retinoid signalling molecule, TRIM16, is repressed during squamous cell carcinoma skin carcinogenesis in vivo and reduces skin cancer cell migration in vitro. J Pathol 2012;226(3):451-62.

Czech-Sioli M, Gunther T, Therre M, Spohn M, Indenbirken D, Theiss J, et al. High-resolution analysis of Merkel Cell Polyomavirus in Merkel Cell Carcinoma reveals distinct integration patterns and suggests NHEJ and MMBIR as underlying mechanisms. PLoS Path 2020;16(8).

Houben R, Shuda M, Weinkam R, Schrama D, Feng H, Chang Y, et al. Merkel cell polyomavirus-infected Merkel cell carcinoma cells require expression of viral T antigens. J Virol 2010;84(14):7064-72.

Houben R, Dreher C, Angermeyer S, Borst A, Utikal J, Haferkamp S, et al. Mechanisms of p53 restriction in Merkel cell carcinoma cells are independent of the Merkel cell polyoma virus T antigens. J Invest Dermatol 2013;133(10):2453-60.

Leonard JH, Dash P, Holland P, Kearsley JH, Bell JR. Characterisation of four Merkel cell carcinoma adherent cell lines. Int J Cancer 1995;60(1):100-7.

Reichrath J, Rafi L, Rech M, Mitschele T, Meineke V, Gartner BC, et al. Analysis of the vitamin D system in cutaneous squamous cell carcinomas. J Cutan Pathol 2004;31(3):224-31.

Rheinwald JG, Beckett MA. Tumorigenic keratinocyte lines requiring anchorage and fibroblast support cultured from human squamous cell carcinomas. Cancer Res 1981;41(5):1657-63.

Rosen ST, Gould VE, Salwen HR, Herst CV, Le Beau MM, Lee I, et al. Establishment and characterization of a neuroendocrine skin carcinoma cell line. Lab Invest 1987;56(3):302-12.

Van Gele M, Leonard JH, Van Roy N, Van Limbergen H, Van Belle S, Cocquyt V, et al. Combined karyotyping, CGH and M-FISH analysis allows detailed characterization of unidentified chromosomal rearrangements in Merkel cell carcinoma. Int J Cancer 2002;101(2):137-45.

Verhaegen ME, Mangelberger D, Weick JW, Vozheiko TD, Harms PW, Nash KT, et al. Merkel cell carcinoma dependence on bcl-2 family members for survival. J Invest Dermatol 2014;134(8):2241-50.
